# Identification of relevant drugable targets in diffuse large B-cell lymphoma using a genome-wide unbiased CD20 guilt-by association approach

**DOI:** 10.1371/journal.pone.0193098

**Published:** 2018-02-28

**Authors:** Mathilde R. W. de Jong, Lydia Visser, Gerwin Huls, Arjan Diepstra, Marcel van Vugt, Emanuele Ammatuna, Rozemarijn S. van Rijn, Edo Vellenga, Anke van den Berg, Rudolf S. N. Fehrmann, Tom van Meerten

**Affiliations:** 1 Department of Hematology, University Medical Center Groningen, University of Groningen, Groningen, the Netherlands; 2 Department of Pathology and Medical Biology, University Medical Center Groningen, University of Groningen, Groningen, the Netherlands; 3 Department of Medical Oncology, University Medical Center Groningen, University of Groningen, Groningen, the Netherlands; 4 Department of Hemato-Oncology, Medical Center Leeuwarden, Leeuwarden, the Netherlands; European Institute of Oncology, ITALY

## Abstract

Forty percent of patients with diffuse large B-cell lymphoma (DLBCL) show resistant disease to standard chemotherapy (CHOP) in combination with the anti-CD20 monoclonal antibody rituximab (R). Although many new anti-cancer drugs were developed in the last years, it is unclear which of these drugs can be safely combined to improve standard therapy without antagonizing anti-CD20 efficacy. In this study, we aimed to identify rituximab compatible drug-target combinations for DLBCL. For this, we collected gene expression profiles of 1,804 DLBCL patient samples. Subsequently, we performed a guilt-by-association analysis with *MS4A1* (CD20) and prioritized the 500 top-ranked CD20-associated gene probes for drug-target interactions. This analysis showed the well-known genes involved in DLBCL pathobiology, but also revealed several genes that are relatively unknown in DLBCL, such as WEE1 and PARP1. To demonstrate potential clinical relevance of these targets, we confirmed high protein expression of WEE1 and PARP1 in patient samples. Using clinically approved WEE1 and PARP1 inhibiting drugs in combination with rituximab, we demonstrated significantly improved DLBCL cell killing, also in rituximab-insensitive cell lines. In conclusion, as exemplified by WEE1 and PARP1, our CD20-based genome-wide analysis can be used as an approach to identify biological relevant drug-targets that are rituximab compatible and may be implemented in phase 1/2 clinical trials to improve DLBCL treatment.

## Introduction

Diffuse Large B-cell lymphoma (DLBCL) is the most common type of Non-Hodgkin lymphoma (NHL). Standard immunochemotherapy consisting of cyclophosphamide, doxorubicin, vincristine, and prednisolone combined with the anti-CD20 monoclonal antibody rituximab (R-CHOP) results in a cure rate of 60% [1]. However, 40% of patients have refractory or relapsing disease and their prognosis is poor [2]. Unfortunately, since the introduction of rituximab two decades ago, all efforts to intensify chemotherapy or develop next generations anti-CD20 antibodies failed to improve their survival [3–5]. For these patients, there is an unmet need to improve standard treatment for DLBCL.

The B-cell receptor (BCR) complex, with the CD20 protein—a product of the *MS4A1* gene—as a part of the BCR signalosome [6], is recognized as an important pathway that drives tumor growth and survival of various B-cell NHLs [7,8]. It has been demonstrated that DLBCL shows the highest basal phosphorylation levels of the BCR complex compared to other B-cell malignancies [9], and that the ongoing antigenic engagement of self-antigens on the BCR is required for tumor survival in activated B-cell (ABC) subtype DLBCL [10]. Emerging data from clinical trials indicate that blocking kinases downstream of the BCR has substantial anti-lymphoma activity. For example, inhibition of BTK, PI3K and SYK through ibrutinib [11,12], idelalisib [13], and fostamatinib [14,15], respectively, has been shown to be effective in follicular lymphoma, mantle cell lymphoma (MCL), and chronic lymphocytic leukemia (CLL). The efficacy of rituximab depends on CD20 clustering within the BCR, whereby rituximab also activates complement in a BCR-dependent manner [16]. In addition, CD20 ligation with monoclonal antibodies on NHL cell lines downregulates important components of the BCR signaling pathway [17,18]. Indeed, kinase inhibitors downstream of the BCR have been shown to interfere with the activity of rituximab [19–22]. Therefore, it is preferred to identify new drug targets for DLBCL outside the context of the CD20/BCR-signalosome.

In the present study, we aimed to identify therapeutic targets for combination therapy in DLBCL, which would be likely to improve treatment outcome without antagonizing the efficacy of rituximab. We therefore collected a large compendium of DLBCL gene expression profiles (GEPs) from the public domain and performed a guilt-by-association analysis with *MS4A1*. Subsequently, after the identification of the well-known but also several unknown DLBCL genes in association with CD20, we prioritized the top-ranked genes for drug-target interaction. Then, as an example, we confirmed high protein expression of two new target genes, WEE1 and PARP1, in DLBCL patient samples. As a next step we combined clinically available inhibiting drugs for these targets with rituximab, which resulted in improved DLBCL cell killing.

## Materials and methods

### Data acquisition and sample processing and quality control

Publicly available raw microarray expression data of DLBCL samples were extracted from the Gene Expression Omnibus (GEO) [23]. The analysis was confined to the Affymetrix HG-U133A (GPL96) and Affymetrix HG-U133 Plus 2.0 (GPL570) platforms.

Non-corrupted raw data CEL files were downloaded from GEO for the selected samples. To identify samples that have been uploaded to GEO multiple times we generated a MD5 (message-digest algorithm 5) hash for each individual CEL file. Before these MD5 hashes were generated we converted all CEL files to the GCOS XDA binary file format (version 4), which was done using the Affymetrix Power Tools (version 1.15.2) apt-cel-convert tool. A MD5 hash acts like a unique fingerprint for each individual file and duplicate CEL files will have an identical MD5 hash. After removal of duplicate CEL files, pre-processing and aggregation of CEL files was performed with RMAExpress (version 1.1.0) by applying the robust multi-array average (RMA) algorithm, using the latest Affymetrix GeneChip Array CDF layout files REF. Principal Component Analysis (PCA) on the sample correlation matrix was used for quality control. The first principal component (PCqc) of such an expression microarray correlation matrix nearly always describes a constant pattern that dominates the data, explaining around 80–90% of the total variance, which is independent of the biological nature of the sample being profiled. The correlation of each microarray expression profile with this PCqc can be used to detect outliers, as arrays of lesser quality will have a lower correlation with the PCqc. We removed samples that had a correlation R < 0.8. To minimize false positive or negative associations due to batch effects (different platforms and experiments) we calculated association statistics within meta-analysis batches. The combination of platform identifier (GPL number, i.e. GEO platform accession number) and experiment identifier (GSE number, i.e. GEO experiment accession number) were defined a meta-analysis batch. Meta-analysis statistic and p-values were calculated according to the generic inverse method with fixed effect model. To assess the degree of multiple testing, we performed this meta-analysis within a multivariate permutation test with 1000 permutation, a false discovery rate of 1% and a confidence level of 99%. For a detailed description we refer to our previous publication [24].

### CD20 (MS4A1) guilt-by-association analysis

Probes representing *MS4A1* were collapsed according to the mean. Next, we used mRNA signals to determine the association of each gene with the expression pattern of *MS4A1*. The association was determined by the Pearson correlation coefficient. Gene set enrichment analyses (GSEA) were performed on the 500 top-ranked *MS4A1*-associated probes (390 unique genes). The 390 *MS4A1* co-expressed genes were uploaded to Enrichr [25], and several gene set databases were consulted (KEGG, Wiki pathways, Biocarta, NCI Nature, Panther and GO biological process). To annotate a single gene to only one biological pathway, we manually marked single genes to 9 different biological pathways (BCR signaling, cytoskeleton regulation, DNA repair and cell cycle, histone modification, immune regulation, metabolism, protein processing, RNA processing, signaling protein (not further specified)).

### Target prioritization

The 390 *MS4A1*-associated genes were analyzed in the drug-gene interaction database (DGidb) [26]. Next, by means of manual curation utilizing Pubmed, clinicaltrials.gov, and the websites of the American Society of Hematology, European Hematology Association, American Society of Clinical Oncology, and the European Society of Medical Oncology, we excluded the identified genes for which anti-neoplastic drugs had been previously investigated in clinical trials with DLBCL patients or already approved for clinical use in DLBCL.

### Cell lines and culture conditions

DLBCL cell lines OCI-ly3, U-2932, SUDHL4 and SC-1 (all obtained from Deutsche Sammlung from Microorganism und Zellculturen, Braunschweig, Germany), SUDHL2 (obtained from American Type Culture collection, Manassus, Virginia, US) and Epstein-Barr virus transformed lymphoblastoid cells (LCL (LCL-1, LCL-2), immortalized from healthy volunteers, anonymized, obtained from A. van den Berg, University Medical Center Groningen [27]) were cultured in RPMI1640 (Lonza BioWhittaker, Walkersville, MD, USA) with 10% Fetal Bovine Serum (FBS; HyClone Thermo Scientific, Waltham, MA, USA), and DLBCL cell lines SUDHL5, SUDHL6 and SUDHL10 in RPMI1640 with 20% FBS. All cell lines were cultured at 37°C with 5% CO_2_ in a humidified atmosphere and in 1% Penicillin-Streptomycin (Lonza BioWhittaker) and 1% Glutamine (Lonza BioWhittaker). The identity of our cell lines was checked periodically by STR profiling.

### Western blot, patient material and immunohistochemistry

Cells were washed with PBS and lysed in RIPA buffer (50mM Tris/ 150mM NaCl/ 2.5mM Na2EDTA/ 1% Triton X-100, 0.5%mM sodium deoxycholate/0.1% SDS in dH_2_0) with 1mM phenylmethanesulphonyl fluoride for 30–45 minutes on ice. Protein concentration was determined using the Pierce^™^ BCA Protein Assay Kit (#23227; Thermo Scientific, Waltham MA, USA). Samples were loaded at 40μg per lane and electrophoresis and blotting was performed according to standard protocols. Staining with primary antibodies for anti-WEE1 (1:200, sc-5285 (B11), Santa Cruz Biotechnology, Dallas TX, USA), anti-phospho-CDC2 (Tyr15) (10A11) (1:1000, #4539, Cell Signaling Technology, Danvers, MA, USA), anti-phospho-Histone H2AX (Ser139) (1:1000, clone JBW301, Merck Milipore, Temecula, CA, USA) and PARP1 (1:1000, #9542, Cell Signaling Technology, Danvers, MA, USA) was done overnight and staining for GAPDH (1:20,000; sc-47724 (0411), Santa Cruz Biotechnology, Dallas TX, USA) was done for one 1 hour at 4°C.

Randomly selected primary formalin fixed paraffin (FFPE) tissue from our anonymous tissue repository (Pathology, University Medical center Groningen) was used of 16 primary DLBCL cases. The study protocol was consistent with international ethical and professional guidelines (the Declaration of Helsinki and the International Conference on Harmonization Guidelines for Good Clinical Practice). The use of anonymous rest material is regulated under the code for good clinical practice in the Netherlands. Informed consent was waived in accordance with Dutch regulations.

Immunohistochemistry (IHC) was performed on FFPE tissue according to standard protocols with appropriate positive and negative controls (based on manufacturer’s instructions). FFPE tissue of 16 randomly selected DLBCLpatients was used. We used the following antibodies: anti-WEE1 (1:200, antigen retrieval with 10mM TRIS/ 1mM EDTA pH9 for 15 min at 120°C, one hour incubation at room temperature, Santa Cruz Biotechnology, Dallas TX, USA) and anti-PARP-1 (1:1000, antigen retrieval with 0.1M TRIS-HCL pH9 for 15 min at 120°C, incubation O/N at 4°C, Biorbyt, Cambridge, UK).

### CD20 flowcytometry

A total of 0.1 x 10^6^ cells were incubated with anti-CD20 (Clone B-Ly1 (R7013), Dako, Glostrup Municipality, Denmark) for 30 minutes on ice in the dark. After washing with 1% BSA in PBS cells were resuspended in 2% paraformaldehyde (Sigma) and analyzed for CD20 expression (mean fluorescence intensity (MFI)) with flow cytometry. To study the effect of PARP1 and WEE1 inhibition on CD20 expression levels, we determined CD20 expression levels with flow cytometry after AZD1775 (WEE1 inhibition) and olaparib (PARP1 inhibition) treatment after 48 hours. For WEE1 inhibition, 0.2 μM AZD1775 for SUDHL6, SUDHL10 and SC-1 was used, and 1 μM AZD1775 for U2932. For olaparib 20 μM was used for SUDHL6, 50 μM for SUDHL10 and SC-1, and 100 μM for U9232.

### Flow cytometry based cytotoxicity assays

A total of 0.1 x 10^6^ cells were pre-incubated with the inhibitor AZD1775 (WEE1 inhibitor, Selleckchem, Houston, TX, USA) for 48 hours at 37°C. After this pre-incubation 0 or 10 μg/mL rituximab with 5% plasma (pooled plasma from 5 donors; Sanquin, the Netherlands) was added for 1 hour at 37°C. Next, cells were washed with 1% BSA in PBS and propidium iodide (Sigma, St. Louis MO, United States) was added for assessment of cell viability via flow cytometry (FACSCalibur, BD Biosciences, Franklin Lakes NJ, United States). Data were analyzed with Winlist 3D (Verity Software house, Topsham ME, USA). Cell lines were determined rituximab-sensitive when > 90% still have propidium iodide uptake upon rituximab treatment.

### AZD1775 and olaparib dose optimization

The optimal concentration window for AZD1775 and olaparib was determined in rituximab sensitive and insensitive cell lines with flowcytometry assays as described above. AZD1775 was titrated in a range from 0.001 μM to 10 μM and olaparib in a range from 1 μM to 10.000 μM.

### Statistical methods

All statistical analysis with respect to survival analysis and *in vitro* assays were undertaken using Graphpad PRISM software as detailed in Supplementary Methods. P-values <0.05 were considered significant.

## Results

### Data acquisition

**Fig 1:**
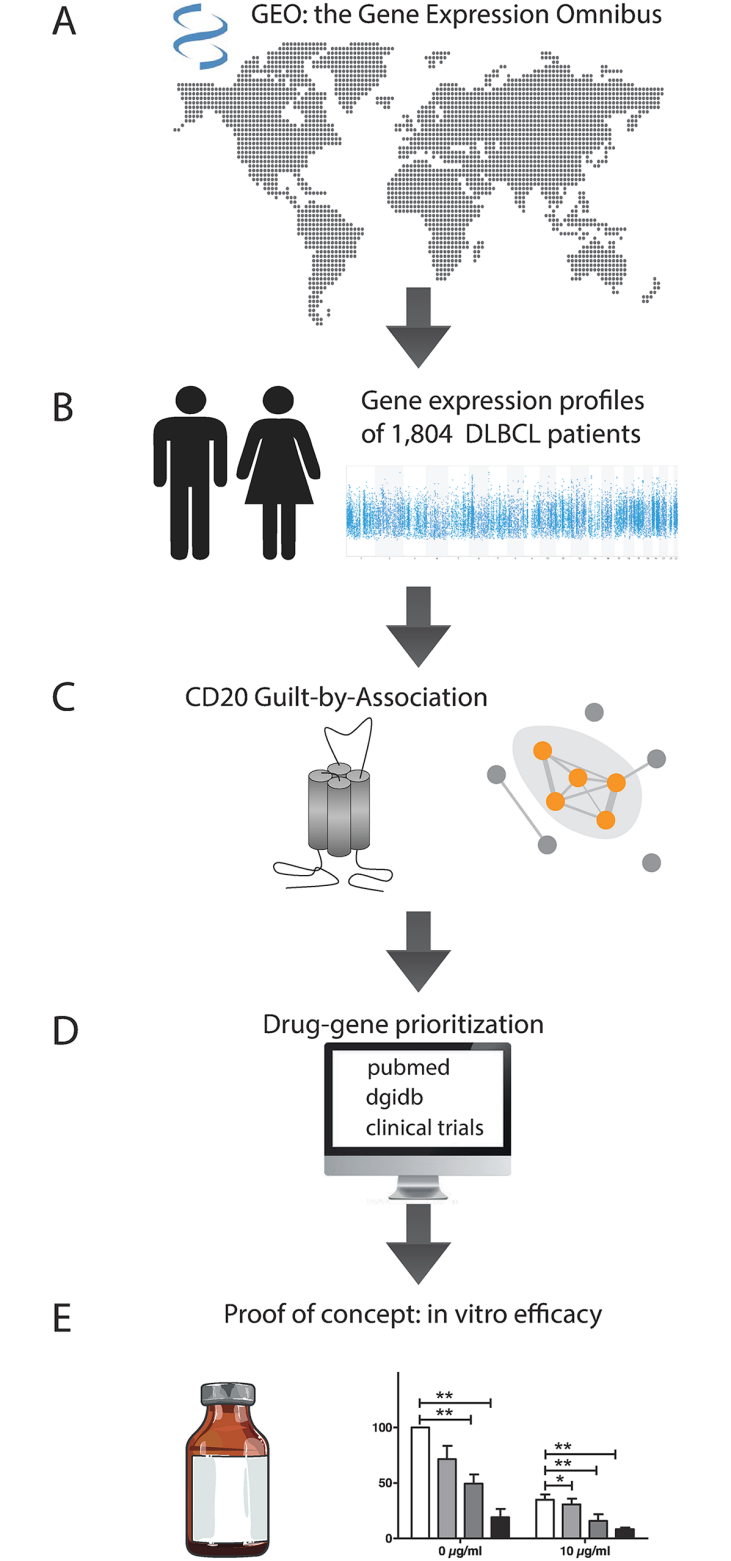
Work flow of the study. (A+B) 1804 Gene expression profiles (GEP) of patients with Diffuse Large B-cell Lymphoma from 20 studies were collected from the gene expression omnibus (GEO). (C) CD20 (gene: MS4A1), as a central protein in B-cell receptor (BCR) signaling and key target for the treatment of DLBCL, was chosen to perform a guilt-by-association analysis. Genes outside the context of BCR signaling (indicated by the grey dots) were chosen for drug-gene prioritization. (D) The Drug Gene Interaction database (DGIdb), Pubmed and clinicaltrials.gov were used to identify drug-gene targets that were not clinically studied in DLBCL before. (E) Two drug-gene targets were chosen for proof-of-concept in vitro studies.

Gene expression profiles of 1,804 DLBCL patients were collected from 20 studies (S1 Table). For all patients meta-data were also included (Fig 1). The majority of the DLBCL expression profiles originated from biopsies of lymph nodes (99%). For 93% of the cases a GEP-based cell-of-origin (COO) was provided, with 35% of the patients being classified as ABC DLBCL, 49% as Germinal Center B-cell (GCB) DLBCL, and 15% as unclassified DLBCL. Treatment data were available for 52% of the patients of which the majority (67%) received R-CHOP, and 33% received CHOP or an Acute Lymphoblastic Leukemia-like regimen. DLBCL patient characteristics are shown in Table 1.

**Table 1:** Patient characteristics of the 20 collected DLBCL studies.

clinical data	Number (and %) of available data	Characteristics of available clinical data
**Age (years)**	**981 (54.5%)**	
**range**		2–94 year
**median**		57.5 years
**Sex**	**988 (54.8%)**	
**Male**		437 (44.2%)
**Female**		551 (55.8%)
**Ann Arbor**	**670 (37.1%)**	
**I**		140 (20.9%)
**II**		175 (26.2%)
**III**		161 (24.0%)
**IV**		192 (28.7%)
**IPI**	**570 (31.6%)**	
**0**		66 (11.8%)
**1**		163 (28.6%)
**2**		160 (27.1%)
**3**		110 (19.3%)
**4**		60 (10.5%)
**5**		11 (1.9%)
**Tissue**	**1796 (99.5%)**	
**Lymph node**		1788 (99.5%)
**Other**		8 (0.4%)
**Treatment**	**1113 (61.5%)**	
**CHOP**		259 (23.3%)
**R-CHOP**		799 (71.9%)
**Other**		54 (4.9%)
**Additional radiotherapy**	**158 (8.8%)**	37 (23.4%)
**Outcome**	**1016 (56.3%)**	
**Cell-of-origin**	**1682 (93.2%)**	
**Activated B-cell**		592 (35.2%)
**Germinal Center B-cell**		830 (49.3%)
**Unclassified**		260 (15.5%)
**MYC rearrangment**	**283 (15.7%)**	
**MYC-neg**		157 (55.5%)
**IG-MYC**		103 (36.4%)
**Non-IG MYC**		23 (8.1%)
**Non-IG translocation BCL-2**	**286 (15.9%)**	
**BCL-2**		44 (15.4%)
**Non-IG BCL-2 expression**	**245 (13.6%)**	
**BCL-2 (pos)**		166 (67.8%)
**Non-IG translocation BCl-6**	**283 (15.7%)**	
** BCL-6**		41 (14.5%)
**Non-IG BCL-6 expression**	**231 (12.8%)**	
**BCL-6 (pos)**		191 (82.7%)

### MS4A1 guilt-by-association

**Fig 2:**
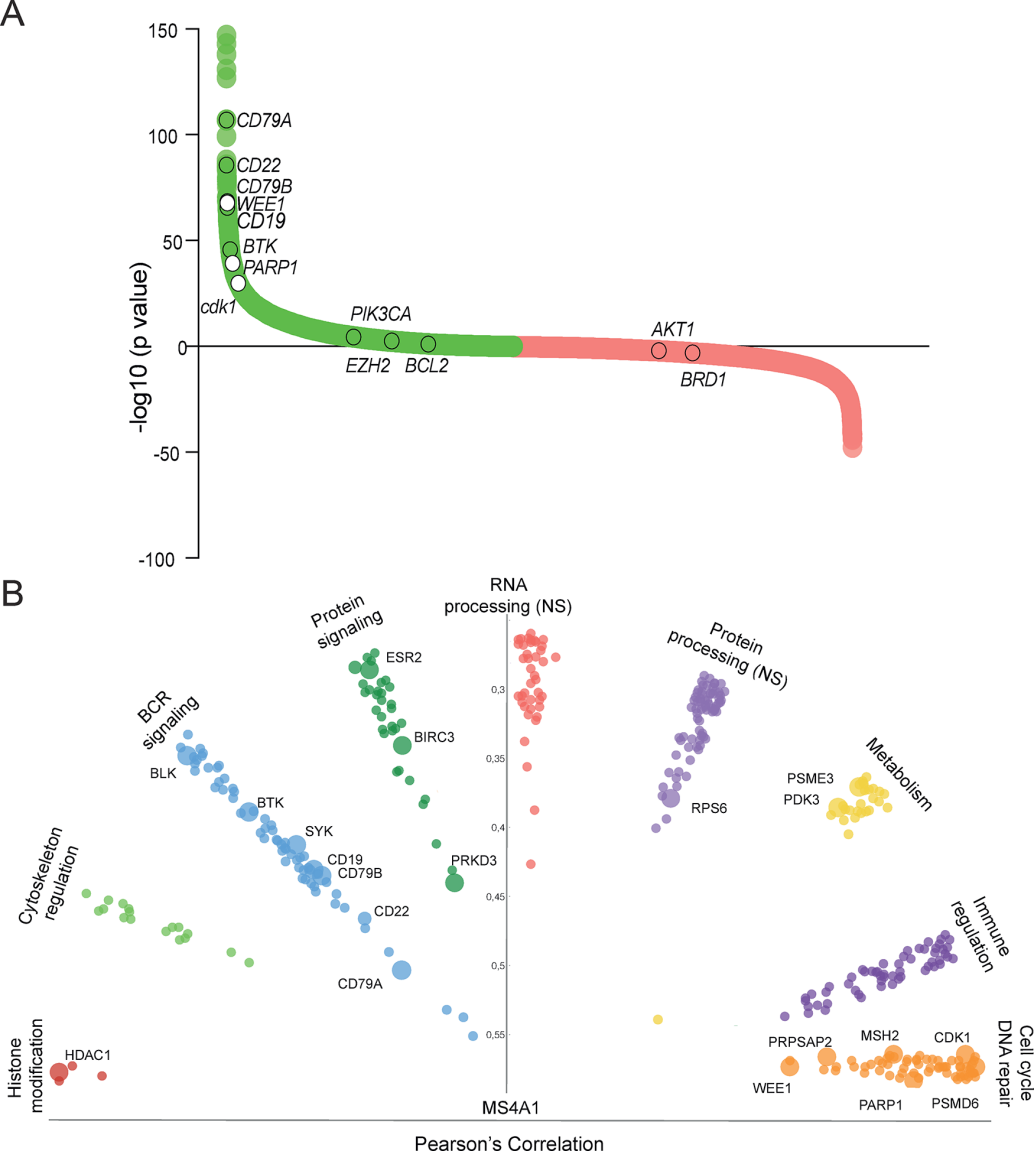
MS4A1 guilt-by-association analysis. (A) Pearson’s correlation plot of MS4A1 Guilt-by-Association of gene expression profiles of 1,804 DLBCL patient samples. In green, genes significantly positively associated with MS4A1, and in red, genes negatively associated with MS4A1. Several known and unknown genes in DLBCL are annotated in white (*MS4A1*-associated genes) and clear circles (drugable targets involved in clinical trials for diffuse large B-cell Lymphoma, but not highly associated with *MS4A1*). (B) The 500 top-ranked *MS4A1* probes (representing 390 genes) were classified into 9 biological subgroups. This plot depicts genes within the subgroups associated to *MS4A1* (Pearson correlation). The big dots represent genes for which clinical inhibitors are available.

To identify genes with similar expression patterns as *MS4A1* we performed a guilt-by-association analysis. We identified 5,355 probes representing 3,893 unique genes that were significantly associated with MS4A1 (FDR 1%, CI 99%) (Fig 2A and S2 Table). As expected, expression of several genes involved in BCR signaling such as *CD79a*, *CD79b* and *CD22* was highly associated with *MS4A1*. For several of these genes, clinically-approved drugs are available and used to treat other types of cancer (Fig 2A). Fig 2A also shows targets that are under clinical evaluation for DLBCL, but for which expression is not associated with *MS4A1*, such as *PIK3CA*, *BCL-2* or *AKT1*. Gene set enrichment analyses (GSEA) of the 500 top-ranked MS4A1-associated probes—representing 390 protein-coding genes—demonstrated a significant over-representation of the BCR signaling pathway according to multiple GSEAs with different gene set databases (e.g. KEGG p = 7.7x10^-9^, Wiki pathways p = 1.8x10^-18^, Biocarta p = 1.7x10^-6^, S2A–S2F Table). To summarize the results of the GSEAs with different gene set databases, we annotated the 390 MS4A1 co-expressed gene set to 9 different biological pathways. Besides the well-known BCR signaling genes and immune regulation genes, other pathways included DNA repair and cell cycle, cytoskeleton regulation, metabolism and histone modification (S4 Table). Correlation of the individual MS4A1-associated genes categorized by biological pathway is shown in Fig 2B. These 390 MS4A1 co-expressed genes include multiple potential targets for DLBCL treatment.

### Target prioritization of *MS4A1*-associated genes

Next, the 390 *MS4A1*-associated gene set was prioritized for drug-gene interactions, to identify targets for which clinically-grade drugs are already available. At least 50 genes had one reported drug-target interaction (S5 Table). Various genes belonging to the BCR signaling pathway were identified, such as like *BTK*, *CD19*, *LYN*, and *SYK*, which can be targeted with ibrutinib, SAR3419, ponatinib, and fostamatinib, respectively. In addition, we identified targets that interact with anti-neoplastic drugs that are currently used in treatment of DLBCL (*e*.*g*. *DHFR* interaction with methotrexate). We also observed targets that are involved in cellular energy metabolism interacting with non-cancer drugs (*e*.*g*. *PRKAB1* with metformin, and *PPP1CA* with vitamin E). In addition, HDAC1 (panobinostat, belinostat, vorinostat, romidepsin), *PSMD3* and *PSMD6* (both carfilzomib) were identified as potential drugs for DLBCL treatment. These drugs are currently under clinical investigation in DLBCL. In Table 2, we summarize the identified drug-target combinations that, to our knowledge, have not been clinically studied in DLBCL patients, and do not interfere with the BCR signalosome. These drugs could potentially be introduced in clinical studies to improve DLBCL patient survival. The potential targets include DNA repair genes and cell cycle, such as *PARP1*, *WEE1*, *CDK1*, which can be targeted by olaparib, AZD1775 and dinaciclib respectively. Other genes are *ESR2*, (targeted by tamoxifen), *PRKD3* (targeted by momelotinib), and *BIRC3* (targeted by AT406). As proof-of-concept of our drug-discovery strategy, we selected *WEE1* and *PARP1*, involved in cell cycle and DNA repair for further preclinical investigations.

**Table 2:** Drug-gene target prioritization.

Gene	Location	Protein	Protein Function	Inhibitor	Clinical Use Inhibitor
BIRC3	11q22	baculoviral IAP repeat containing 3	inhibits apoptosis by binding to tumor necrosis factor receptor-associated factors	AT-406	Ovarium cancern/ Acute myeloid Leukemia
PARP1	1q41-q42	poly (ADP-ribose) polymerase 1	repair of single-stranded DNA breaks	olaparib	Mammae and prostate cancer
PRKD3	2p21	protein kinase D3	Binding of diacylglycerol and phorbol esters	momelotinib	Myelofibrosis
RP56 / IMPG2	3q12.2-q12.3	interphotoreceptor matrix proteoglycan 2	organization of the interphoto-receptor matrix and may promote the growth	PX-866	Non-small-cell lung cancer
WEE1	11p15.4	WEE1 G2 checkpoint kinase	tyrosine kinase, catalyzes the inhibitory tyrosine phosphorylation of CDC2/cyclin B kinase	AZD1775 / MK1775	Solid tumors
ESR2	14q23.3	estrogen receptor 2 (ER beta)	protein forms homo- or hetero-dimers that interact with specific DNA sequences to activate transcription	tamoxifen	mammacarcinoma
CKD1	10q21.2	Cyclin-dependent kinase 1	Ser/Thr protein kinase family and catalytic subunit protein kinase complex known as M-phase promoting factor	Dinaciclib	Chronic Lymfocytic Leukemia and multiple myeloma
PDK3	Xp22.11	pyruvate dehydrogenase kinase, isozyme 3	nuclear-encoded mitochondrial multienzyme complex that catalyzes the overall conversion of pyruvate to acetyl-CoA and CO_2_	CPI-613	advanced hematologic malignancies
MAP3K1	5q11.2	mitogen-activated protein kinase kinase kinase 1, E3 ubiquitin protein ligase	serine/threonine kinase and is part of transduction cascades, including the ERK and JNK kinase pathways as well as the NF-kappa-B pathway	AZD8330	advanced malignancies

### Relevance of *WEE1* and *PARP1* mRNA expression in DLBCL treatment

**Fig 3:**
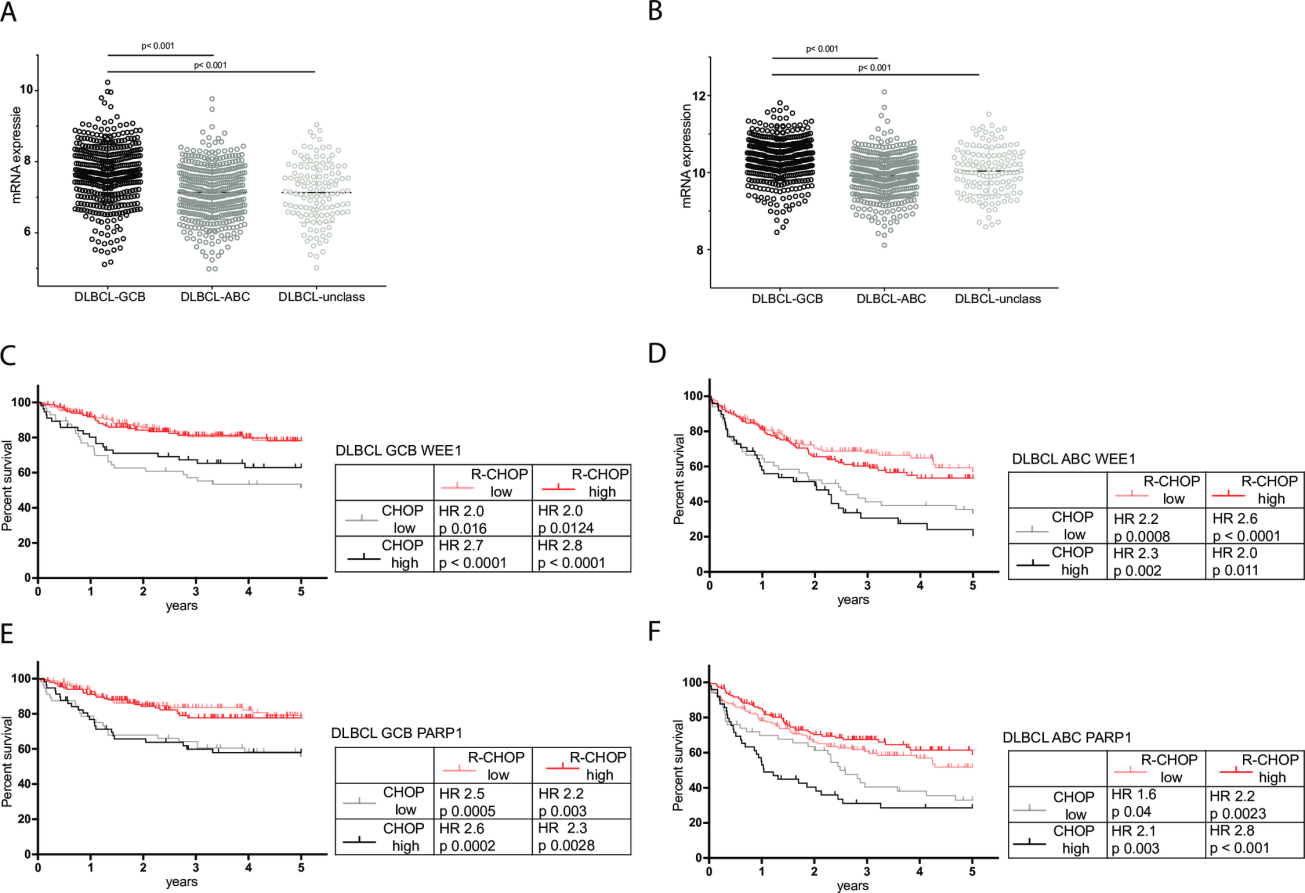
Expression levels of *WEE1* and *PARP1* in different DLBCL subgroups and in relation to anti-CD20 therapy with or without standard chemotherapy. (A) *WEE1* and (B) *PARP1* mRNA expression levels in Germinal Center B-cell (GCB, black), Activated B-cell (ABC, dark grey), and unclassified (light grey) Diffuse Large B-cell Lymphoma (DLBCL) samples. Overall survival for patients with DLBCL-GCB (C) and DLBCL-ABC (D) with low and high *WEE1* expression treated with CHOP or R-CHOP, and overall survival for DLBCL-GCB (E) and DLBCL-ABC (F) patients with low and high *PARP1* expression treated with CHOP or R-CHOP. Shown in the tables provided are the hazard ratios of adding anti-CD20 therapy with rituximab to standard chemotherapy (cyclophosphamide, doxorubicin, vincristine, and prednisone (CHOP)). Log-rank testing was used to test whether the curves are statistically different and to calculate the hazard ratio’s.

For both *WEE1* and *PARP1*, mRNA expression was significantly higher within the GCB DLBCL subtype compared to ABC and unclassified subtypes (Kruskall-Wallis p< 0.001, Fig 3A and 3B). Survival and treatment data were available for 872 patients (R-CHOP and CHOP). Improved overall survival was observed in patients treated with R-CHOP compared to CHOP in DLBCL patients in all COO subgroups (S1 Fig). The addition of rituximab to CHOP was markedly more beneficial in GCB-DLBCL patients with high WEE1 expression than in patients with low WEE1 expression (Hazard Ratio (HR) of 2.8, CI 1.5–5.1, p = 0.001 vs HR 2.0. CI 1.0–3.8, p = 0.016) (Fig 3C). For ABC-DLBCL patients with low or high *WEE1* expression we observed no differences in the addition of rituximab to CHOP chemotherapy, respectively (HR of 2.2, CI 1.3–3.6, p = 0.0008 vs HR 2.0. CI 1.2–3.3, p = 0.001) (Fig 3D).

In GCB-DLBCL there were no differences in survival HRs for the addition of rituximab to CHOP in patients with high or low PARP1 expression (high PARP1: HR 2.3, CI 1.4–4.8, p = 0.003 vs low PARP1 HR 2.6, CI 1.4–4.8, p = 0.0005, Fig 3E). However, addition of rituximab to CHOP was markedly more beneficial with respect to survival in ABC-DLBCL patients with high PARP1 expression than in patients with low PARP expression (HR 2.8, CI 1.6–4.7. p = 0.001 vs HR 1.6 CI 0.9–2.5 p = 0.04) (Fig 3F). These data show that the additional effect of rituximab to CHOP may also be associated with the expression level of WEE1 and PARP1.

### WEE1 and PARP1 protein expression and targeting of WEE1 and PARP1 kills DLBCL cell lines

**Fig 4:**
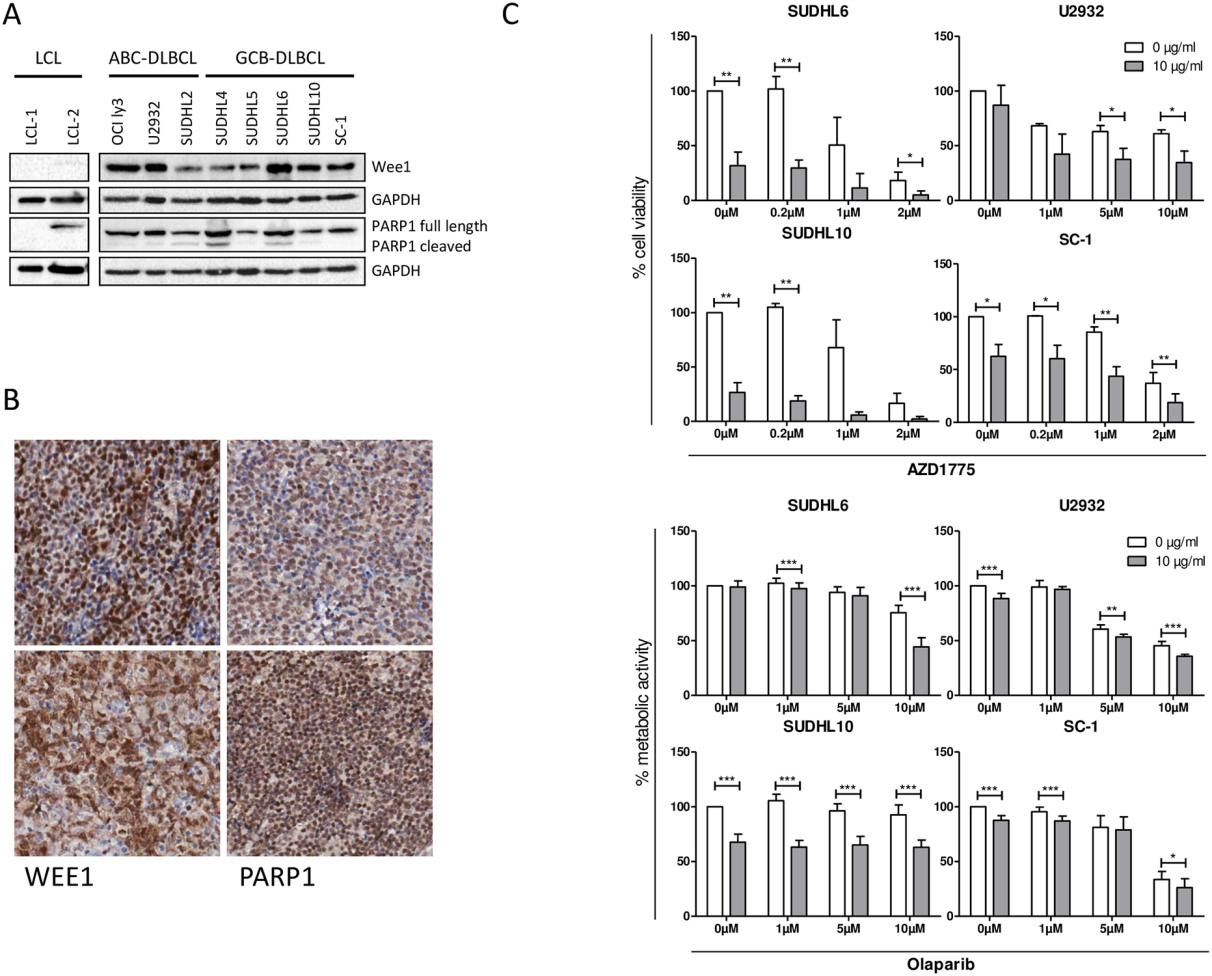
Protein expression of WEE1 and PARP1 in DLBCL and in in vitro killing assays. (A) Western blot results for Wee1, PARP1 in eight DLBCL cell lines. Two LCL cell lines are shown as normal B-cell controls. (B) Immunohistochemistry of Wee1 (left column) and PARP1 (right column) on DLBCL patient samples. Both Wee1 and PARP1 showed a nuclear staining pattern. (C) Cytotoxicity assays of the WEE1 inhibitor AZD1775 with or without rituximab in two rituximab sensitive and two resistant cell lines: SUDHL6 (rituximab sensitive, RS), SUDHL10 (RS), U2932 (rituximab insensitive, RI) and SC-1 (RI). Shown is the normalized live population (propidium iodide negative population) of three independent experiments. Student T-test was used to demonstrate significance (*) p<0.05/ (**) p<0,005. (D) Resazurin metabolic activity assay with the PARP1 inhibitor olaparib with or without rituximab in the above-mentioned cell lines. Shown is the normalized metabolic activity of three independent analyses. Student T-test was used to compare samples without inhibitor treatment. Significant (*) p< 0.05 / (**) p<0,005 / p<0.001 (***).

Immunoblotting revealed WEE1 and PARP1 expression in all eight DLBCL cell lines, and not in control LCL cells (Fig 4A). In FFPE tissue samples both WEE1 and PARP1 showed a nuclear staining pattern in tumor cells. WEE1 was expressed in 14 out of 16 cases (78%) and PARP1 in 15 out of 16 cases (94%), for both WEE1 and PARP1 the percentage of positive cells and protein intensity levels differed between patient samples (Fig 4B). This indicates that WEE1 and PARP1 are expressed at the protein level in DLBCL, both in DLBCL cell lines and primary cases.

Next, we tested the effect of WEE1 and PARP1 inhibitors on DLBCL cell lines as single agent and in combination with rituximab. Single agent rituximab killing assays demonstrated that 4 of the 8 DLBCL cell lines were sensitive to rituximab treatment, corresponding to CD20 expression levels (S2A and S2B Fig). We selected 2 rituximab-sensitive (RS, SUDHL6 and SUDHL10) and 2 rituximab-insensitive (RI) cell lines (U2932 and SC-1) for further preclinical investigation. As a single agent, increasing concentrations of the WEE1 inhibitor AZD1775 strongly reduced cell viability in RS and RI cell lines after 48 hours (Fig 4C), without influencing CD20 expression levels (S2C Fig). Combining AZD1775 with rituximab showed a significant additional decline of cell survival in all tested cell lines (Fig 4C). In the DLBCL cell line SUDHL6 (RS), adding rituximab to a concentration of 2 μM AZD1775 decreased cell viability from 18% to 5% (p = 0.0311) compared to AZD1775 alone, for SUDHL10 (RS), adding rituximab to a concentration of 0.2 μM AZD1775 decreased cell viability from 105% to 18% (p = 0.0015) compared to AZD1775 alone, for the U2932 cell line (RI), cell viability decreased from 62% to 37% at 5 μM (p = 0.00154) compared to AZD1775 alone, and for SC-1 (RI), a concentration of 2 μM AZD1775 plus rituximab decreased cell viability from 36% to 18% (p = 0.0039). Similar results were obtained when WEE1 inhibition with rituximab was tested in the resazurin metabolic activity assay (S2D Fig).

PARP1 inhibition by clinically obtained olaparib dose levels had limited single agent activity (Fig 4C). However, in cell viability assays combining 10 μM olaparib with rituximab in SUDHL6 (RS) resulted in an additional decline in cell viability (75% to 44% (p<0.001)), for the SUDHL10 cell line (RS), a concentration of 1 μM olaparib with rituximab decreased cell viability from 105% to 63% (p<0.001), for the U2932 cell line (RI), cell viability decreased from 60% to 53% at 5 μM (p = 0.003), and for the SC-1 cell line (RI), a concentration of 10 μM olaparib plus rituximab decreased cell viability from 33% to 26% (p = 0.03) (Fig 4C).

In conclusion, the combination of WEE1 or PARP1 inhibition with rituximab resulted in enhanced cytotoxicity and reduced cell viability in 3 out of 4 tested almost all DLBCL cell lines. The added effect of the WEE1 or PARP1 inhibitors with rituximab was independent of rituximab sensitivity.

## Discussion

In this study, we performed a large meta-analysis on the transcriptomic data of 1,804 DLBCL patient samples to identify drug-target combinations for improvement of standard DLBCL immunochemotherapy (R-CHOP). We therefore took CD20, which is part of the BCR signalosome and a key target in DLBCL treatment, as the central protein to perform a guilt-by-association analysis. By employing CD20 for guilt-by-association we aimed to find targets with similar expression patterns to CD20. We focused on the associated genes as therapeutic targets for DLBCL. Co-expression does not necessarily indicate a direct relation or interaction with CD20, but was used for selection of promising targets. Guilt-by-association analysis has been used in cancer research to identify biomarkers. However as a therapeutic purpose, guilt-by-association has been used only to identify targets in defined pathways, such as cancer metabolism [28]. In the present study, we used this method for the first time to identify targets in relation to a single gene—CD20 –which is a central molecule for current treatment regimens of DLBCL patients. This guilt-by-association approach may also be applied more generally in future studies to improve drug combinations for other types of cancer and any starting gene with a central role in standard therapies.

We selected the top 500 associated probes, corresponding to 390 protein-encoding *MS4A1*-associated genes. All well-known genes to be actively involved and expressed in DLBCL were present, including for instance BTK as a target for ibrutinib in current DLBCL clinical trials. In addition, we identified many genes for which the pathogenetic relevance in the context of DLBCL is still unknown (Table 2). From this list, candidate drug-targets were selected when not involved in BCR signaling or currently already under clinical study in DLBCL. Moreover, only clinical-grade inhibiting drugs from the treatment of other (solid) malignancies were selected to accelerate their application in clinical trials. The choice for clinically approved drugs also circumvents the problem of a worldwide lack of a proper mouse model to study the effect of rituximab *in vivo*. The human Fc region of the chimeric IgG1 antibody rituximab lacks the ability to activate the murine complement (CDC) and effector cells (ADCC) [29,30], thereby limiting the study of relevant rituximab-drug combinations in a murine or xenogeneic setting.

Our selection revealed multiple targets which were more strongly associated with CD20 than other well-known targets in DLBCL. We therefore consider them to be of high potential for direct combination with current DLBCL treatment. Examples are CDK1 (cell cycle; targeted by dinaciclib, PRKD3 (signaling protein; targeted by momelotinib), WEE1 (replication checkpoint kinase; targeted by AZD1775) and PARP1 (DNA repair; targeted by olaparib).

For primary investigation we chose *WEE1* and *PARP1*. Although neither of these genes have been investigated in DLBCL in combination with rituximab, both WEE1 and PARP1 have clinically approved inhibiting drugs and have been studied extensively *in vivo*. Both are currently used in clinical trials for several (solid) cancers, including cervical cancer, ovarian cancer, breast cancer, lung cancer, adenocarcinoma and gliomas (ClinicalTrials.gov). Another important reason for our interest in these two genes was based on DLBCL pathophysiology. DLBCL originates from normal B-cells due to aberrant effects of somatic hypermutation and class-switch recombination machinery during the germinal center reaction, which results in chromosomal breaks leading to oncogenic transformation of B cells [31,32]. There is a crucial role for DNA damage response (DDR) and repair proteins during the germinal center reaction [33] and high expression of DNA damage response proteins have been demonstrated in DLBCL patient cases [34]. Since DLBCL is a tumor with high levels of DNA damage, targeting proteins involved in DDR and damage repair, such as WEE1 and PARP1, is a rational choice for therapy in DLBCL.

WEE1 is a replication checkpoint kinase that prevents the onset of mitosis in cells that have incompletely replicated or have damaged genomes. In case of DNA damage, WEE1 indirectly arrests the cells at the G2/M checkpoint, allowing time for repair or resulting in cell death [35]. Targeting WEE1 with AZD1775 in patients with a diversity of chemo-refractory solid tumors demonstrated single agent activity [36]. Targeting WEE1 with AZD1775 in combination with the CHK1 inhibitor PF-00477736 resulted in cell killing and destabilization of the oncogenic transcription factor MYC in DLBCL and was strongly synergistic in mantle cell lymphoma [37,38]. Moreover, great potential has been shown for WEE1 inhibition in combination with cell cycle arresting chemotherapeutics such as doxorubicin and cytarabine [39]. Our results show that WEE1 is highly expressed in DLBCL patient specimen. In addition, we demonstrated that the combination of the WEE1 inhibitor AZD1775 and rituximab resulted in additive cytotoxicity for all tested DLBCL cell lines, also in the rituximab-insensitive cell lines.

PARP1 is well-known for its role in repairing DNA single strand breaks, and is thought to accumulate at sites of damage, inducing chromatin remodeling and attracting DNA repair factors [40]. PARP inhibitors have been mainly used in a setting of defective double strand break repair (DSBR), as PARP inhibition leads to double stranded breaks, which causes synthetic lethality in a DSBR defective background. To this extent, PARP1 inhibition has proven to be successful when used in DDR deficient tumors such as BRCA1- or BRCA2-deficient breast cancer, ATM-deficient colorectal cancer [41], ATM-deficient lung cancer [42], TP53/ATM-deficient MCL [43], IGH/MYC-induced BRCA2 deficient Burkitt lymphoma [44] [and PTEN/TP53-deficient prostate cancer [45]. In DLBCL, *TP53* mutations are found in 21–24% of cases and are inversely correlated with survival [46,47]. Moreover, PARP1 is known for its role in NF-kB activation [48] contributing to inflammation and carcinogenesis. Therefore, targeting PARP1 in a setting of high genomic instability, as seen in DLBCL, and high NF-kB activation, as seen in the ABC type DLBCL [49], is an understandable choice. Our results demonstrate that PARP1 is highly expressed in DLBCL patient samples. Interestingly, this finding is supported by the recently published PARP1-targeted PET imaging approach which can differentiate malignant from inflamed lymph nodes in DLBCL [50].

The combination of the PARP inhibitor olaparib and rituximab enhanced cytotoxicity in all 4 DLBCL cell lines tested, which all carried mutations in the *TP53* gene. Consequently, combining PARP1 inhibitors with current therapy could improve survival of patients with mutant TP53. Recently, the potential synergistic effects of combining WEE1 and PARP1 inhibition in acute leukemia revealed also a potential synergistic effect, creating a double-hit model by increasing DNA damage and preventing DNA damage repair [51].

A potential bias of our approach might have been the selection of only high-quality mRNA samples. For this reason we performed survival analyses for the different COO DLBCL groups and for CHOP versus R-CHOP treated DLBCL patients. These results were similar to survival data as reported in the literature. The addition of rituximab to CHOP chemotherapy seems more beneficial in GCB-DLBCL with high WEE1 expression compared to low WEE1 expression. This might be explained by the correlation of WEE1 with CD20 expression level as observed in our guilt-by-association analysis, as patients with low CD20 expression also have inferior survival [30,52]. For PARP1, our data showed that patients with a relatively high PARP1 expression in ABC-DLBCL benefitted the most from the addition of rituximab to CHOP chemotherapy. This suggests an additional effect of PARP1 response in the ABC subtype patients to rituximab. We hypothesize that this might be explained by the continuous activation and essential role of NF-κB in ABC-subtype DLBCL. Rituximab directly inhibits subunits of the NF-κB pathway [53] and might therefore lead to accumulation of more damage in ABC-type DLBCL that depends on high PARP1 expression for repair and NF-κB activation.

In conclusion, a genome wide analysis of *MS4A1* (CD20) guilt-by-association and drug-target prioritization has been able to identify potential relevant drug-targets to combine with and improve DLBCL treatment. For the identified genes *WEE1* and *PARP1* clinically approved inhibitory drugs showed improved DLBCL cell killing when combined with rituximab. Our approach may be used as a fast-track approach to direct the use of clinically approved agents in future phase I/II trials to improve standard DLBCL treatment.

## Supporting information

S1 Table GSE accession numbers. Series indentifier and GPL platform numbers that were used in this study, including correspsonding references.
(XLSX)

S2 Table CD20 guilt_by_association. Shown are the first 500 *MS4A1*-associated gene probes with corresponding gene symbol, gene title and chromosomal location by Pearson’s correlation with confidence interfals (CI).
(XLSX)

S3 Table S3A-F Table enrichment analysis. Different gene set databases were consulted (KEGG, Wiki pathways, Biocarta, NCI Nature, Panther and GO biological process).
(XLSX)

S4 Table CD20 guilt-by-association subgroup analysis. The first 500 *MS4A1*-associated gene probes with corresponding gene name were grouped according to biological pathway.
(XLSX)

S5 Table Prioritized drug-gene targets. List of drug-gene interactions for the first 500 *MS4A1*-associated gene probes, with corresponding pubmed IDs..
(XLSX)

S1 Fig Overall survival for patients with diffuse large B-cell lymphoma (DLBCL). (A), Germinal Center B-Cell (GCB) DLBCL (B), Activated B-cell (ABC) DLBCL (C), and unclassified DLBCL (D) treated with CHOP or R-CHOP. Log-rank testing was used to test whether the curves are statistically different (* p-value < 0.0001, ** p-value 0.003). Abbreviation: R-CHOP: rituximab, cyclophosphamide, doxorubicin, vincristine and prednisone.
(TIF)

S2 Fig In vitro activity of rituximab, AZD1775 and olaparib. (A) CD20 expression level of 8 different Diffuse Large B-cell Lymphoma (DLBCL) cell lines. The cell-of-origin is indicated of each individual cell line. (B) The in vitro susceptibility of the DLBCL cell line to rituximab in the presence of human complement. (C) Western blot results of WEE1, PARP1 and yH2AX protein expression of SUDHL16 and SUDHL10 treated for 24 hours with 1 μM AZD1775 or 250 μM Olaparib. (D) Resazurin metabolic activity assay of the WEE1 inhibitor AZD1775 with or without rituximab in two rituximab sensitive and two resistant cell lines: SUDHL6, SUDHL10, U2932, and SC-1. Shown is the normalized metabolic activity of three independent analyses. Data was analyzed with student T-test as compared to sample without inhibitor treatment. Significant (*) p< 0.01/ (**) p<0,001/ (***) p < 0.0001.
(TIF)
