# Supplementary figures and images for: Identification of relevant drugable targets in diffuse large B-cell lymphoma using a genome-wide unbiased CD20 guilt-by association approach
Source: PLoS One. 2018 Feb 28;13(2):e0193098. doi: 10.1371/journal.pone.0193098 (PMC5831110; doi:10.1371/journal.pone.0193098)

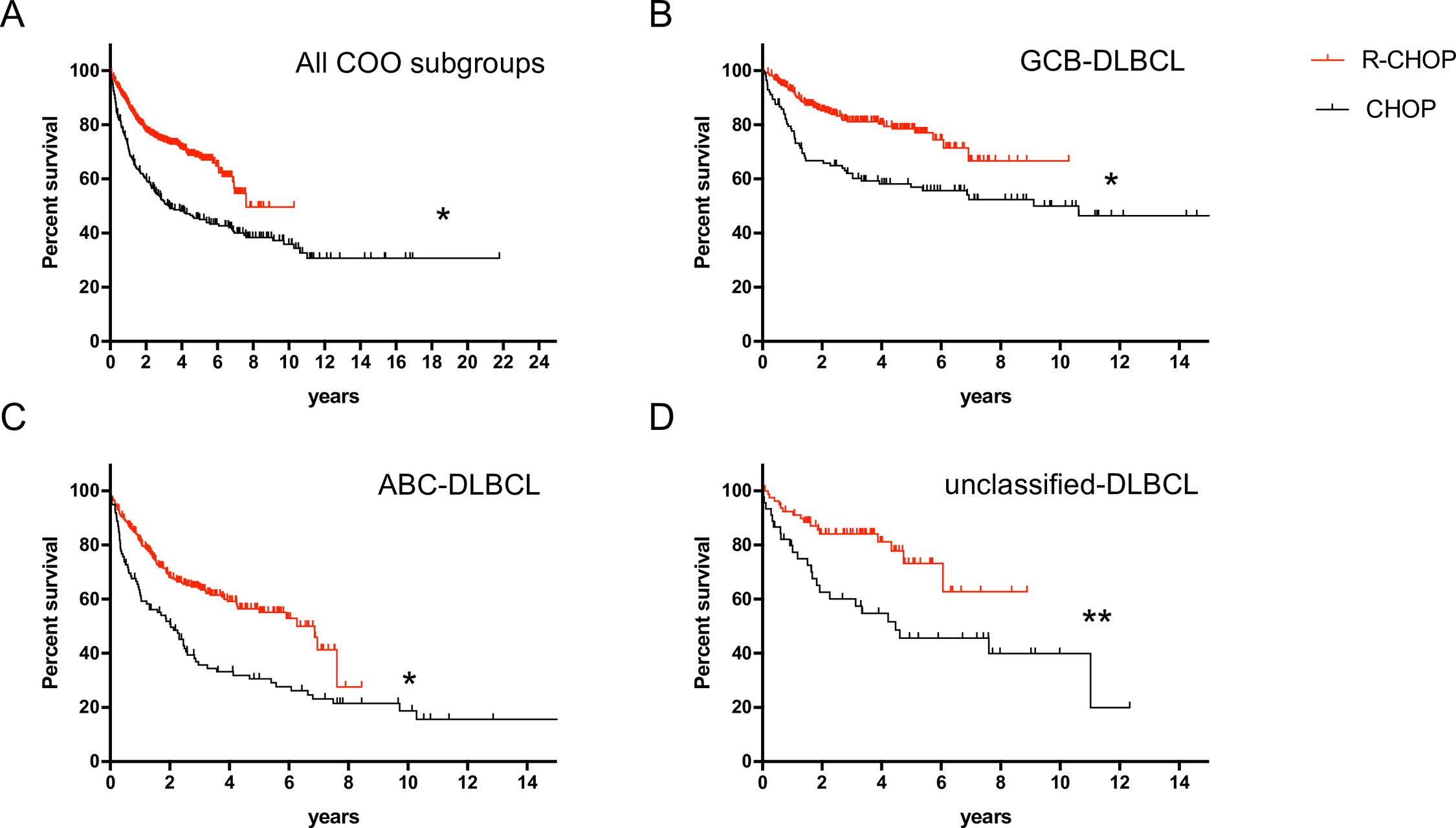

Supplement: S1 Fig — (A), Germinal Center B-Cell (GCB) DLBCL (B), Activated B-cell (ABC) DLBCL (C), and unclassified DLBCL (D) treated with CHOP or R-CHOP. Log-rank testing was used to test whether the curves are statistically different (* p-value < 0.0001, ** p-value 0.003). Abbreviation: R-CHOP: rituximab, cyclophosphamide, doxorubicin, vincristine and prednisone. (TIF) [file pone.0193098.s006.tif]

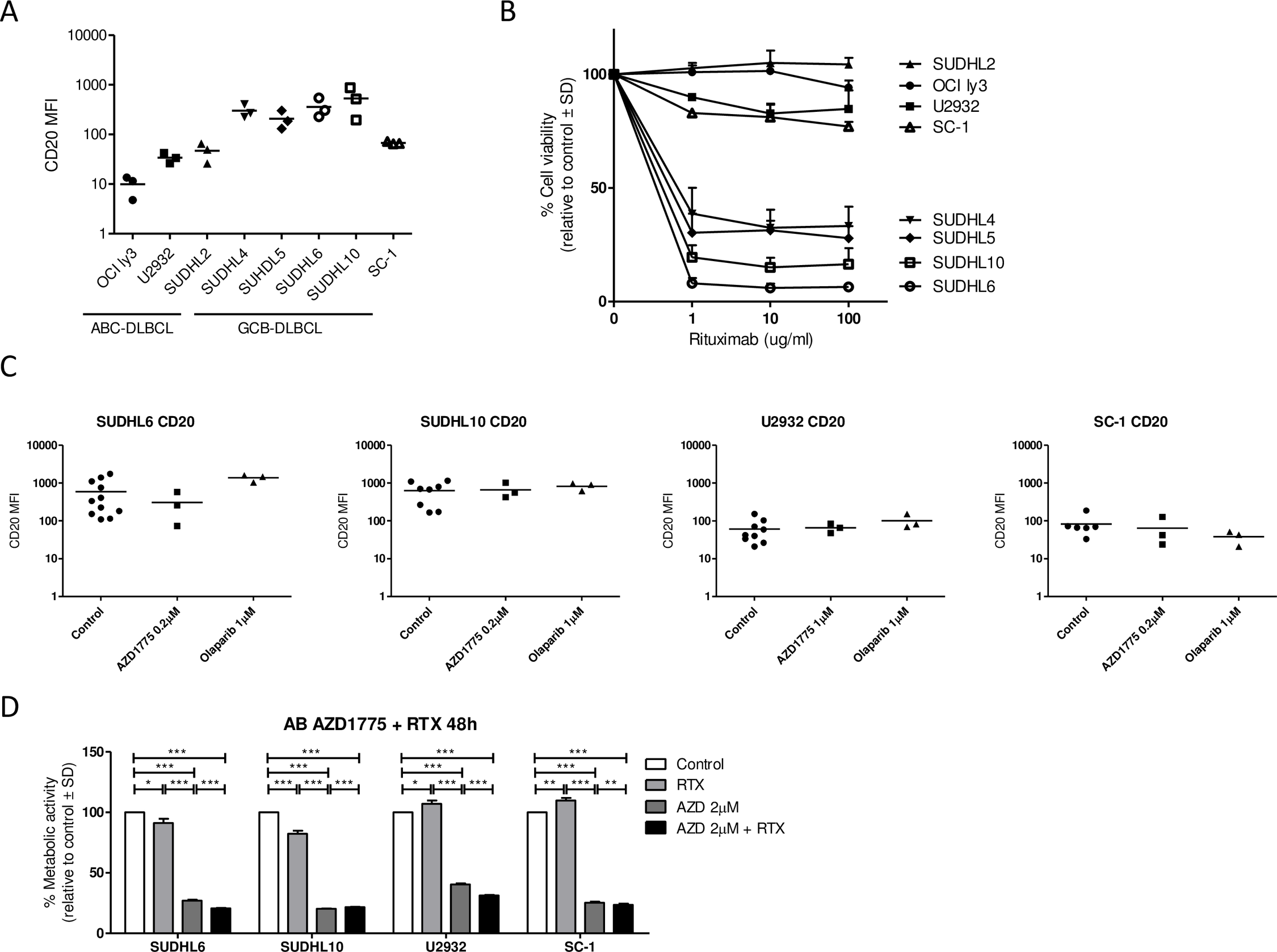

Supplement: S2 Fig — (A) CD20 expression level of 8 different Diffuse Large B-cell Lymphoma (DLBCL) cell lines. The cell-of-origin is indicated of each individual cell line. (B) The in vitro susceptibility of the DLBCL cell line to rituximab in the presence of human complement. (C) Western blot results of WEE1, PARP1 and yH2AX protein expression of SUDHL16 and SUDHL10 treated for 24 hours with 1 μM AZD1775 or 250 μM Olaparib. (D) Resazurin metabolic activity assay of the WEE1 inhibitor AZD1775 with or without rituximab in two rituximab sensitive and two resistant cell lines: SUDHL6, SUDHL10, U2932, and SC-1. Shown is the normalized metabolic activity of three independent analyses. Data was analyzed with student T-test as compared to sample without inhibitor treatment. Significant (*) p< 0.01/ (**) p<0,001/ (***) p < 0.0001. (TIF) [file pone.0193098.s007.tif]
